# Electronic Systems Diagnosis Fault in Gasoline Engines Based on Multi-Information Fusion

**DOI:** 10.3390/s18092917

**Published:** 2018-09-03

**Authors:** Jie Hu, Tengfei Huang, Jiaopeng Zhou, Jiawei Zeng

**Affiliations:** 1 Hubei Key Laboratory of Advanced Technology for Automotive Components, Wuhan University of Technology, Wuhan 430070, China; tenfi@whut.edu.cn; 2 Hubei Collaborative Innovation Center for Automotive Components Technology, Wuhan 430070, China; 3 Shanghai E-propulsion Auto Technology Co. Ltd., Shanghai 210800, China; whutzhou@126.com; 4 Cummins East Asia Research & Development Co. Ltd., Wuhan 430070, China; jw_zeng@126.com

**Keywords:** gasoline engine, electronic control system, fault diagnosis, multi-information fusion

## Abstract

The rapid development of electronic techniques in automobile has led to an increase of potential safety hazards, thus, a strong on-board diagnostic (OBD) system is desperately needed. To solve the problem of OBD insensitivity to manufacture errors or aging faults, the paper proposes a novel multi information fusion method. The diagnostic model is composed of a data fusion layer, feature fusion layer, and decision fusion layer. They are based on the back propagation (BP) neural network, support vector machine (SVM), and evidence theory, respectively. Algorithms are mainly focused on the reliability allocation of diagnostic results, which come from the data fusion layer and feature fusion layer. A fault simulator system was developed to simulate bias and drift faults of the intake pressure sensor. The real vehicle experiment was carried out to acquire data that are used to verify the availability of the method. Diagnostic results show that the multi-information fusion method improves diagnostic accuracy and reliability effectively. The study will be a promising approach for the diagnosis bias and drift fault of sensors in electronic control systems.

## 1. Introduction

The wide application of on-board electrical and electronic components and subsystems in modern vehicles increases the instability of the system and promotes the booming of the potential safety hazard. To ensure functional safety, the automobile industry has developed its own standard (ISO26262), which defines the functional safety requirements and life-cycle (management, development, production, service, and decommissioning) management for the safety-related components of automobiles in different phases of the safety lifecycle [1]. 

In compliance with the standard, developing a strong on board diagnostic (OBD) system is an effective way. Although the OBD system has made great progress in recognizing the electrical circuit failures, such as the short to ground fault, short to battery fault, and open pin fault, it is still a dead area, which cannot effectively diagnose the fault caused by electric devices’ manufacturer errors or aging, such as the bias fault and drift fault. Furthermore, for the electronic systems of gasoline engine control, it is highly dependent on the availability and accuracy of sensor measurements [2]. For example, once an intake pressure sensor suffers from a bias or drift fault, suitable gas cannot be supplied for the engine, which will lead to instability of the engine speed, fluctuation of the output torque, deterioration of emissions, and degradation of the vehicle drivability. Therefore, an effective diagnosis method is needed to solve this problem.

Diagnosis methods can generally be classified into model based methods and data driven methods. For decades, many researchers pushed the application of model based methods to design fault diagnosis systems, such as Rizzoni [3], Blanke [4], and Ding [5]. The model based method compares the model output with the system operating value to diagnose faults based on the mathematical model, so its diagnosis accuracy relies on the accuracy of the model. Especially for highly complicated systems, like gasoline engines, it is usually hard to get an accuracy model for the system [6]. 

In contrast, data driven methods do not need a mathematical model. Additionally, it only dependss on the measured process data, and can process these massive signals and recognize the health conditions of the machinery automatically [7,8]. Moreover, with the recent boom and development of big data, cloud computing, and artificial intelligence technology, it is easier to obtain sufficient high-quality data and high-speed computing components. These offer abundant oil for the development of the data driven method. As such, nowadays, data driven methods have been widely used to design fault diagnosis and fault detection and isolation systems [9]. 

Data driven methods mainly adopted in the diagnostic area are the the back propagation (BP) neural network, support vector machine (SVM), and evidence theory. Efforts have been made to study these methods. The BP neural network is a kind of non-logic and non-language artificial intelligence approach based on the connection structure, which has many advantages, such as parallel structure, parallel processing, distributed storage, good error tolerance, self-organization, self-learning, and reasoning. In a previous study [10], the BP algorithm was used to detect and diagnose faults of a marine engine cooling system. In another study [11], the intelligent diagnosis of engine faults was realized by using a BP neural network to realize the typical malfunction of a certain truck engine. In another study [12], a BP neural network was used for recognition of acoustic signals, together with the nearest neighbour classifier and the modified classifier. The authors of [13] showed that neural networks can be a very useful tool for solving many scientific and practical problems related to the mining industry. Another previous study [14] contributed to the usage of an artificial neural network as a decisive part in surface roughness prediction. The SVM method was put forward by Vapnik in 1995. It is an effective classification method based on the Vapnik–Chervonenkis (VC) dimension of statistical learning theory and structural risk minimization principle. It can automatically identify the support vector that has a good distinction ability for classification. The authors of [15] used the support vector machine (SVM) method to effectively recognize the turbocharger fault, analyze the reason for the failures, and realize fault prediction and prevent accidents. Li et al. [16] applied symbolic dynamic entropy features to extract features of the gearbox signals and applied the support vector machine to recognize health conditions based on the dynamic characteristics of gearbox signals. Evidence theory is an efficient method that is better than traditional probability theory on grasping the unpredictability and uncertainty of the problem. In addition, it provides the method for evidence synthesized methods and can fuse evidence obtained from various evidence sources. A previous study [17] used the weighted Dempster-Shafer (DS) evidence theory to diagnose engine faults.

When each of them is used separately as a single diagnosis algorithm, the reliability and confidence level of the diagnosis result tends to be low and weak, and there exists the possibility of false diagnosis, especially in highly complicated mechanical systems. Hence, it is necessary to fusion data driven methods to diagnose faults [18]. 

The authors of [19] reviewed the application of multi information fusion in the field of vehicles. A previous study [20] used the information fusion and classification method to diagnose spark plug faults of an internal combustion engine; for a single application of the artificial neural network, the diagnostic accuracy was 67.46%; for a single application of the least squares support vector machine, the diagnostic accuracy was 65.08%; and, after the use of the evidence theory fusion, the classification accuracy reached 98.56%. In another study [21], evidence theory was used as a modeling tool, and information was regarded as relevant evidence, which reflects the quality of the engine. A previous study [22] used the multi information fusion method, which is combined with the artificial neural network and evidence theory, to diagnose faults of a diesel engine. In another paper [23], the multi information of aero engines and artificial intelligence technology were combined to diagnose sensor faults, gas path faults, and mechanical vibration faults. In another study [24], by using neural network and evidence theory, the fault diagnosis of the sensor and actuator for an electronic control engine are made. Another study [25] used the multi information fusion method to diagnose coolant temperature sensor faults and oxygen sensor faults. In a previous study [26], a method for multi-sensor information fusion based on Dempster-Shafer (DS) evidence theory is discussed for fault diagnosis of the aero-engine gas path.

In this paper, a novel multi information fusion method (which combines the BP neural network, SVM, and evidence theory) is proposed to diagnose electronic systems of gasoline engines. In detail, the bias and drift fault of intake pressure sensors will be regarded as the targets to diagnose and verify the feasibility of the method.

The remainder of the paper is organized as follows: The second section introduces the multi information fusion algorithm, and explains the reason why the multi information fusion algorithm can improve the reliability of diagnosis results. The third section establishes the multi information fusion fault diagnosis model, including the data fusion layer model, feature fusion layer model, and decision fusion layer model, and focuses on the reliability allocation of diagnosis results from the data fusion layer and feature fusion layer. The fourth section describes the experiment process, including the fault simulator development and real vehicle experiment. The fifth section uses the multi information model to analyze the acquired experimental data. The last section summarizes the whole paper and proposes future research.

## 2. Multi Information Fusion

The basic principle of multi information fusion, also called data fusion, is to simulate the procedure of human processing information, according to certain fusion rules, with complementary information in space and time. It makes full use of the advantages of diversification, obtains valuable decision-making information, and improves the accuracy of results, with the premise of the consistency of data. The feasibility of the multi information fusion method can be proved by information theory.

It is assumed that $\Theta =\left\{\theta_{1},\theta_{2},\ldots ,\theta_{N}\right\}$ on behalf of the engine running state set. Additionally, the engine running state probability is expressed as $p_{i}=p\left\{\theta =\theta_{i}\right\}$. The entropy, *H*, of the engine running state, θ, indicates the state of uncertainty, as shown in Formula (1).

(1) $$H(\theta )=-\sum_{i=1}^{N}p_{i}\cdot \log p_{i}$$

It is assumed that because $X\in \left\{x_{1},x_{2},\ldots ,x_{m}\right\}$ represents the diagnostic information set, and $X=x_{j}$ is known, the condition entropy and the average conditional entropy of engine operating condition can be calculated, according to Formulas (2) and (3).

(2) $$H(\theta |X=x_{j})=-\sum_{i=1\;}^{N}p(\theta_{i}|x_{j})\cdot \log p(\theta_{i}|x_{j})$$

(3) $$H(\theta |X)=-\sum_{j=1\;}^{M}p(x_{j})\cdot H(\theta |X=x_{j})$$

By computation derivation, it can be inferred that the condition of the engine state would be more than or equal to the conditional entropy. If the engine diagnostic information, *X*, is known, the uncertainty of the running state, *θ*, will be improved.

It is assumed that the mutual information of the engine status and diagnostic information reflects the uncertainty relationship between them. The mutual information calculation formula is expressed as (4).

(4) $$I(\theta ,X)=H(\theta )-H(\theta |X)=\sum_{\theta ,x}p(\theta ,x)\cdot \log\frac{p(\theta ,x)}{p(\theta )\cdot p(x)}$$

When the case engine diagnostic information is known, the greater the mutual information value, the more determined the engine running state, and the more the diagnostic information, *X*, can characterize the running state of the engine. If another engine diagnostic information, *Y*, is added, the mutual information calculation formula would be (5).

(5) $$I(\theta ,X,Y)=H(\theta )-H(\theta |X,Y)=\sum_{\theta ,x,y}p(\theta ,x,y)\cdot \log\frac{p(\theta ,x,y)}{p(\theta )\cdot p(x,y)}$$

From Formula (5), it is known that if the fault diagnostic information increases, the fault certainty will be improved further, and the reliability of diagnosis will be enhanced [27]. Therefore, using the method of multi information fusion can reduce the uncertainty degree effectively and improve the accuracy in the diagnostic process.

## 3. Engine Fault Diagnostic Model

**Figure 1:**
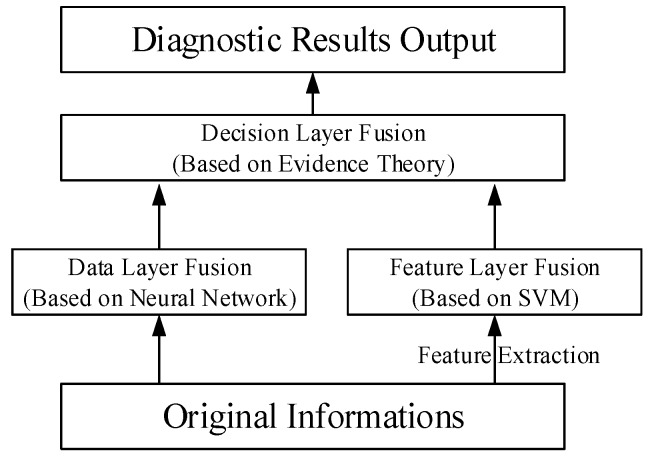
Engine fault diagnostic model based on multi information fusion.

The engine fault diagnosis model based on multi information fusion is built according to the data processing level. The model is composed of the data fusion layer, feature fusion layer, and decision fusion layer, as shown in Figure 1.

### 3.1. Data Layer Fusion Model

In essence, engine fault diagnosis is pattern classification through identifying the running state with operating parameters. At the same time, it is the classification of multiple kinds of faults, which is difficult to diagnose through the physic model. Fortunately, the neural network provides a way. The data fusion layer algorithm can use the BP neural network. Because the neural network has a strong ability to identify and classify the associative memory capacity, multiple-input multiple-output models with complex nonlinear relationships can quickly and accurately achieve learning and training.

**Figure 2:**
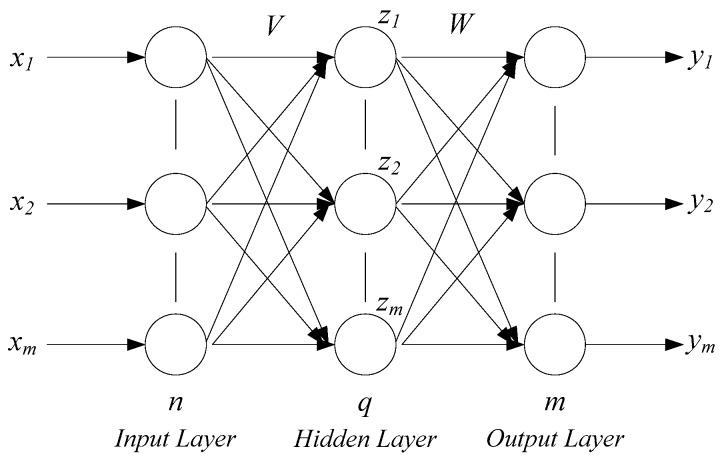
BP neural network structure diagram.

Neural networks usually contain an input layer, hidden layer, and output layer. Its structure diagram is shown in Figure 2 [13]. The input layer and output layer are single simple structures, and the number of nodes is determined by the application characteristics. For the layer number of the hidden layer, many researchers have conducted theoretical analysis and have found that if the number of hidden nodes are enough, the single hidden layer structure can simplify nonlinear function approximation. The number of hidden layer nodes mainly relies on experiences and trials. The excitation function of the BP neural network is usually chosen between the sigmoid function and hyperbolic tangent function. The number of output layer nodes mainly relies on the dimension of the expectation output. 

The general learning process of the BP neural network is divided into two stages, the calculation results forward transfer and error reverse transmission. In the forward transfer phase, sample data are sent from the input layer to the hidden layer for calculation. Then, the BP neural network obtains the calculation results at the output layer. If the difference between the network calculation results and expected results does not meet the design requirements, the BP network will work in the next stage (error back propagation phase), and otherwise the network training is completed. In the error reverse transfer phase, error is decomposed to each layer of neurons, and the weight factor and threshold factor of each neuron is corrected according to the decomposition value [12]. 

**Figure 3:**
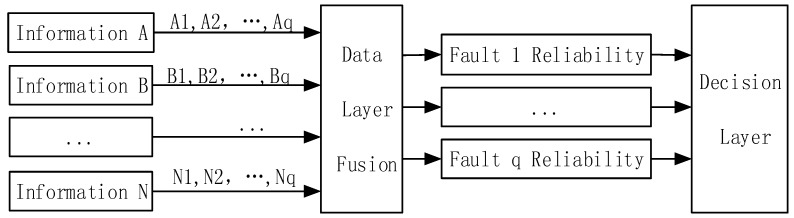
The data layer fusion model based on the BP neural network.

The detailed fault diagnosis process is: Firstly, parameters are sampled with the same engine condition continuously, and ensures the conformity of the acquired parameters in the time aspect. Next, using the normalization method to dispose of the sample parameters, the results are put into the neural network to do the data fusion layer, part of them as training sets and others as test data. Finally, reliability of the diagnosis results is allocated from the data fusion layer, which will be sent to the decision fusion layer to make the final decision. The established data fusion layer model based on the BP neural network is shown in Figure 3.

### 3.2. Feature Fusion Layer Model

In the feature fusion layer, firstly, the multidimensional features of the collected information should be extracted and reduced. Then, they will be regarded as input for decision-making in the higher level fusion for fault diagnosis. The feature fusion layer algorithm is the SVM, which is similar to the neural network. SVM uses feature data corresponding directly to the fault mode, and does not need the support of diagnostic rules, which have lower data quantities, but more feature dimensions [28].

SVM theory assumes that there is a sample set, $\left\{\left(x_{1},y_{1}),\;(x_{2},y_{2}),\;\ldots ,\;(x_{l},y_{l}\right)\right\}$, $x\in R^{D},\;y\in \left\{-1,1\right\}$. Where, *l* is the number of samples, *D* is the number of samples feature, and *y* is the sample patterns. It is also assumed that there are only two kinds of attribute values, ωx+b=0 is the hyperplane, *H*, $\omega x_{i}+b=0$, *y =* 1 is the hyperplane, *H1*, which parallels to the plane of the hyperplane, and the distance between *H* and *H1* is *y =* 1. $\omega x_{i}+b=0$, *y =* −1 is the hyperplane, *H2*, which parallels to the plane of the hyperplane, and the distance between *H* and *H2* is also *y =* −1.

**Figure 4:**
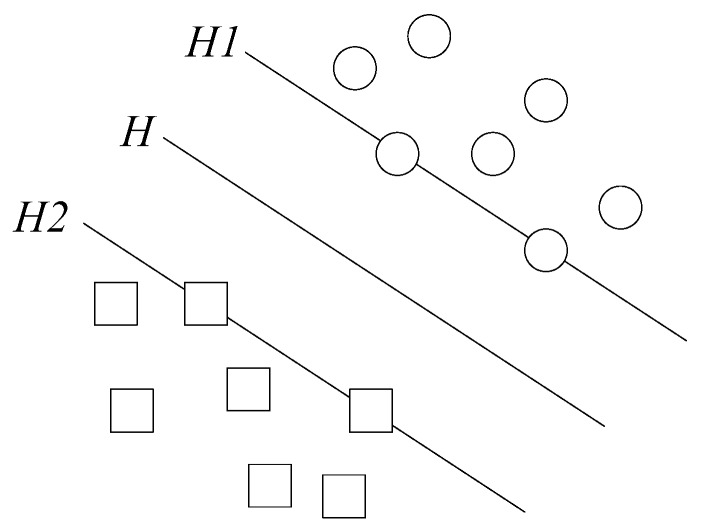
SVM classification diagram.

If the distance between *H1* and *H2* is maximized, then *H* is the optimal hyperplane, and *H1* and *H2* are support vectors of the upper sample data. The classification diagram is shown in Figure 4, where the square points and dots represent two types of data.

**Figure 5:**
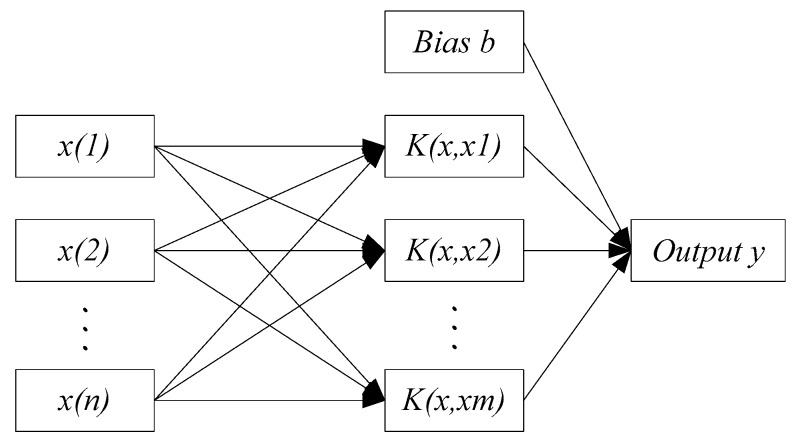
SVM network structure.

**Figure 6:**
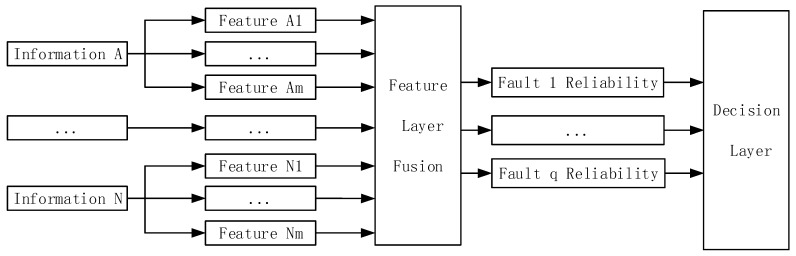
Feature fusion layer model based on support vector machine.

The SVM network structure is shown in Figure 5. *K* is the kernel function, including the linear kernel function, radial basis function, polynomial kernel function, and so on. The established feature fusion layer model based on the support vector machine is shown in Figure 6.

### 3.3. Decision Layer Fusion Model

The acquired reliability of diagnosis results from the data layer and feature layer are low, which means that there exists the problem of false diagnosis in some cases. To improve the diagnostic accuracy and reliability, diagnosis results of the data fusion layer and feature fusion layer will be fused to make decisions in the decision fusion layer. In this paper, D-S evidence theory is used as the algorithm for the decision fusion layer.

D-S evidence theory gets the final decision based on the reliability, *m(A),* of the evidence through analyzing and synthesizing the evidence. The reliability is the degree of belief for the established proposition, *A*. Evidence refers to objective characteristics, personnel subjective experience, and the knowledge that depends on the reliability of the object to be calculated. The essence of the evidence theory is to determine the extent that an unknown object belongs to the identified frame, Θ (which denotes a set that contains every possible solution of a problem, all the elements of it are mutually exclusive), under the condition of the identification frame determined. Set $m:2^{\Theta }\to [0,\;1]$ is the basic reliability allocation in the recognition framework, Θ. According to the D-S evidence theory, the support of an arbitrary assumption is presented by an interval. The lower limit of this interval is called the confidence function, which is defined as:

(6) $$Bel(A)=\sum_{B\subset A\;}m(B),\;\forall A\subseteq \Theta$$

It is assumed that the confidence function, $Bel_{1},Bel_{2},\ldots ,Bel_{n}$, is assigned in the same identification framework. $m_{1},m_{2},\ldots ,m_{n}$ denotes the basic confidence distribution functions in the same recognition frame, Θ. If $Bel_{1}⊕Bel_{2}⊕\ldots ⊕Bel_{n}$ exists, there is a reliability assignment, as shown in Equation (7).

(7) $$\left\{ \begin{array}{l} m(A)=Bel_{1}⊕Bel_{2}⊕\ldots ⊕Bel_{n}=K\sum_{\begin{array}{l} A_{1},A_{2},\ldots ,A_{n}\subset \Theta \;\\ A_{1}\cap A_{2}\cap \ldots \cap A_{n}=A \end{array}}m_{1}(A_{1})\cdot \cdot \cdot m_{n}(A_{n}),\Theta \neq \phi \\ m(A)=Bel_{1}⊕Bel_{2}⊕\ldots ⊕Bel_{n}=0,\Theta =\phi \end{array}\right.$$

Value *K* is the conflict degree, which presents the conflict degree among the evidence, which can be calculated as shown in (8).

(8) $$K=\frac{1}{1-\sum_{\begin{array}{l} A_{1},A_{2},\ldots ,A_{n}\subset \Theta \;\\ A_{1}\cap A_{2}\cap \ldots \cap A_{n}=\phi \end{array}}m_{1}(A_{1})\ldots m_{n}(A_{n})}$$

The above reliability formula is also called synthetic principle of evidence theory, and the final reliability is obtained by the belief function of each evidence. The evidence combination rule offers a comprehensive combination rule of the multiple independent bodies of evidence, and the law has the nature of association. In the body of evidence synthesis, the combination sequence has no effect on the final synthesis results, so evidence can be in any combination.

When 0<K<1, there is a conflict of evidence reliability, but there still exists a consistency. It can be processed according to the evidence combination rule to obtain the synthetic results. In the case of *K =* 1, it means that evidence is completely opposite, and is not in accordance with the evidence rules of evidence synthesis processing. Therefore, it is necessary to calculate the degree of reliability conflict degree and judge whether the fusion diagnosis can be carried out.

The fault diagnostic procedure in the decision fusion layer based on evidence theory is depicted as follows: Firstly, the characteristics of the data fusion layer and feature fusion layer algorithm are united, and the reliability of the data fusion layer and feature fusion layer diagnostic results are allocated. Then, the evidence of the degree of conflict is calculated, and it is determined whether the evidence theory combination rules can be adopted to calculate the reliability of the proposition. Finally, the proposition that has the maximum reliability as output of the decision layer is chosen. In the decision fusion layer, the critical thing is to allocate the reliability of the data fusion layer and diagnosis results of the feature fusion layer.

#### 3.3.1. Reliability Allocation Based on Diagnostic Results of Data Fusion Layer 

According to relevance theory, the basic reliability, $m_{i}(F_{j})$, and uncertainty description, $m_{i}(\Theta )$, can be defined as Formulas (9) and (10), as follows.

(9) $$m_{i}(F_{j})=\frac{C_{i}(F_{j})\;}{\sum_{j}C_{i}(F_{j})+R_{i}}$$

(10) $$m_{i}(\Theta )=\frac{R_{i}\;}{\sum_{j}C_{i}(F_{j})+R_{i}}$$

where, $C_{i}(F_{j})$ is the normalized value of the diagnosis results for the BP neural network in the data fusion layer, and $R_{i}=1-\alpha_{i}\cdot \beta_{i}\cdot \omega_{i}$ represents the diagnosis procedures aggregate uncertainty. The parameter, $\alpha_{i}$, $\beta_{i}$, $\omega_{i}$, can be calculated using (11)–(13).

(11) $$\alpha_{i}=C_{i}(F_{m})-\underset{j\neq m}{\max}\left\{C_{i}(F_{j})\right\},C_{i}(F_{m})=\underset{j}{\max}\left\{C_{i}(F_{j})\right\}$$

(12) $$\beta_{i}=\sqrt{\frac{1}{N-1\;}\sum_{j=1}^{M}{\left(C_{i}(F_{j})-\mu_{i}\right)}^{2}},\;\mu_{i}=\frac{1}{M-1}\sum_{j=0,j\neq m}^{M}C_{i}(F_{j})$$

(13) $$\omega_{i}=\frac{\alpha_{i}\beta_{i}}{\sum_{i=1}^{N}\alpha_{i}\beta_{i}}$$

where, *α_i_* is the difference between the maximum and second largest relevance in evidence, *E_i_*, of the sub proposition, which reflects the reliability of the sub proposition. In addition, the bigger the *α_i_*, the higher the reliability of the sub proposition in the recognition framework, Θ. *β_i_* is the relevance correlation variance with evidence, *E_i_*, in the recognition framework, Θ, of other sub propositions (except the sub proposition that has the biggest relevance with evidence, *E_i_*), which reflects the correlation degree of polymerization for other sub propositions. In addition, the bigger the *β_i_*, the worse the degree of polymerization. *μ_i_* is the relevance mean value (except the sub proposition, which has the biggest relevance with the evidence, *E_i_*) of other sub propositions. *ω_i_* is the weight factor of the evidence, *E_i_*. In the application of fault diagnosis, different evidence bodies have different sensitivities to fault, which makes the difference of the characteristic value of the evidence. Therefore, the weight factor is introduced to construct the reliability distribution function to improve the accuracy of decision results.

#### 3.3.2. Reliability Allocation Based on Diagnosis Results of the Feature Fusion Layer 

Accordingly, using vote results to allocate reliability is characteristic of the feature fusion layer algorithm, which is based on SVM in the decision fusion layer. In the SVM model, the vote numbers in the one to one classification model are counted, then the whole classification numbers are divided, and the basic probability distribution function of the sub proposition is obtained, as shown in formula (14).

(14) $$m(f_{i})=V_{i}/\sum_{i=1}^{n}V_{i}=V_{i}/C_{n}^{2},i=1,2,\ldots ,n$$

where, $f_{i}$ is a type of i, $V_{i}$ is the vote numbers in the whole one to one classification model, *n* is the total type, and $C_{n}^{2}$ is the total classification.

## 4. Fault Simulator Development and Real Vehicle Experiment

### 4.1. Fault Simulator Development

For validating the diagnostic effects of the multi information fusion method repeatedly, a fault simulator was developed. When there is a bias fault or drift fault, related electrical signals change accordingly, so it is feasible to take notice of the normal signal to simulate faults. Bias fault simulation method is to up bias or down bias based on the normal signal, and the drift fault simulation method is to delay the output of the normal signal. Detailed disposal methods are shown as Formulas (15) and (16).

Where, $V_{out}(t)$ is the simulated output signal at time, *t*, $V_{in}(t)$ is the actual output signal at time, *t*, Δa is the bias value, and $t_{a}$ is the delay time.

(15) $$V_{out\;}(t)=V_{in}(t)\pm \Delta a$$

(16) $$V_{out\;}(t)=V_{in}(t-t_{a})$$

**Figure 7:**
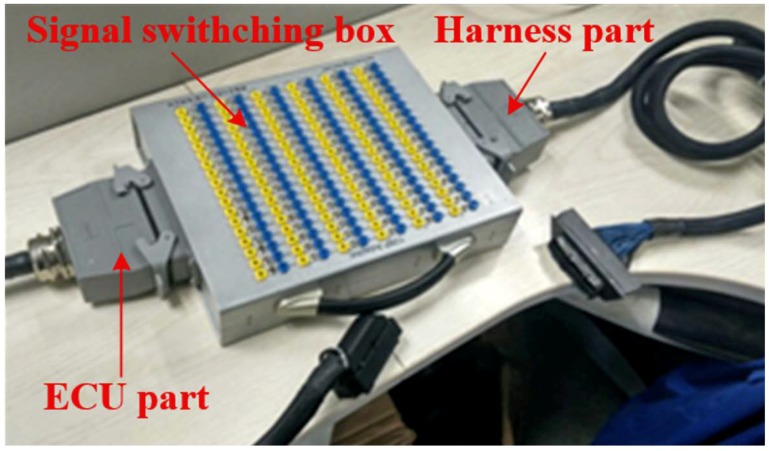
Signal switching box.

The fault simulate experiment is operated under real vehicle conditions. In such condition, it is tough to input intake air pressure signals to the fault simulator directly without the destruction of the harness because the engine electronic control system harness is highly integrated. However, equipment with a specialized signal switching box can avoid this problem. The signal switching box is located between the Electronic Control Unit (ECU) and harness, which is shown in Figure 7.

**Figure 8:**
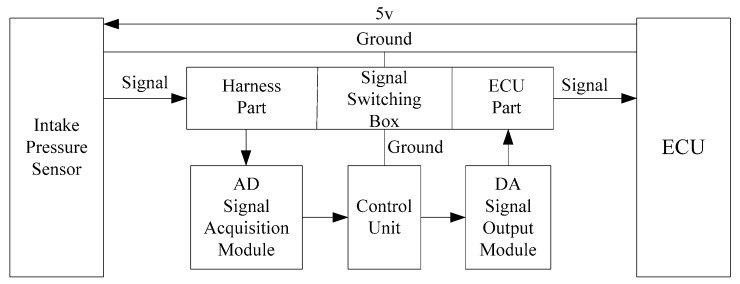
Fault simulator hardware connection figure.

Because the intake air pressure sensor is an analog signal sensor, the fault simulator should be equipped with an analog to digital signal acquisition module, data conversion module, and digital to analog signal output module. The voltage of the intake air pressure sensor variation range is 0–5 V. The Analog-to-Digital (AD) module can transfer the signal voltage from the harness part of the signal switching box directly to the control unit for processing, then output the processed signal to the ECU part of the signal switching box through the DA module. The fault simulator, intake air pressure sensor, and ECU have the same ground, and the intake air pressure sensor 5 V supply voltage directly connects to the ECU. The fault simulator hardware connection is shown in Figure 8. 

**Figure 9:**
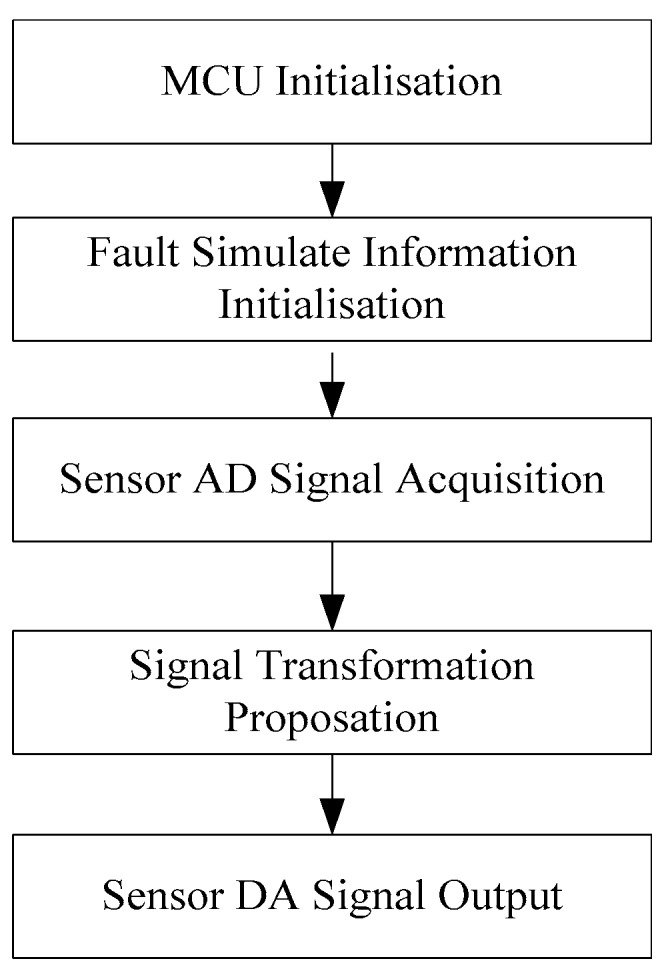
Fault simulator software program flowchart.

The fault simulator software development environment is CodeWarrior IDE. Its running process is shown as follows: Firstly, the microcontroller hardware module, including the AD sampling module, clock module, and Serial Peripheral Interface (SPI) communication module is initialized. Secondly, the fault simulated information is initialized, confirming the simulated fault, including the fault mode selection, signal amplitude selection, and delay time parameter setup. Thirdly, the analog signal is acquired, transferring to the digital value. Then, according to the selected fault mode, the amplitude, delay time and calculation formula, converting the acquired value. Finally, the value to the analog signal, and output to the ECU through the DA module is transferred. The bias calculation formula is the bias signal voltage divided by the sampling accuracy, which is 5 mV. The delay amount calculation formula is the delay time divided by the single instruction running time, which is dependent on the oscillator frequency and frequency division. The fault simulator software program flowchart is shown in Figure 9.

### 4.2. Real Vehicle Experiment

**Figure 10:**
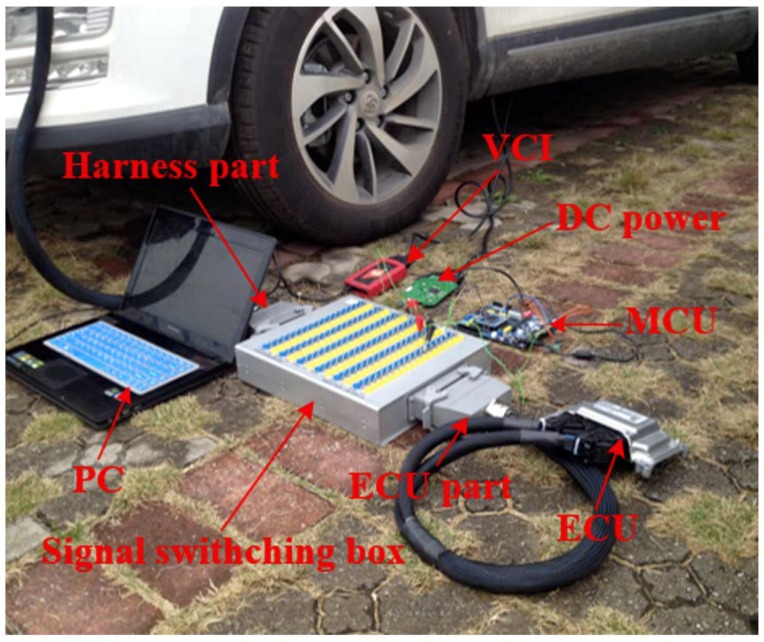
Fault simulated real vehicle experiment.

The sensor bias fault and drift fault can be simulated by the fault simulator. These fault modes include the sensor signal voltage normal, upward bias, downward bias, time delay, and signal voltage loss. A detailed simulated experiment plan is shown in Table 1. The real vehicle experiment is shown in Figure 10. Where, VCI is the vehicle connection interface, which can transfer data between the Controller Area Network (CAN) and PC.

**Figure 11:**
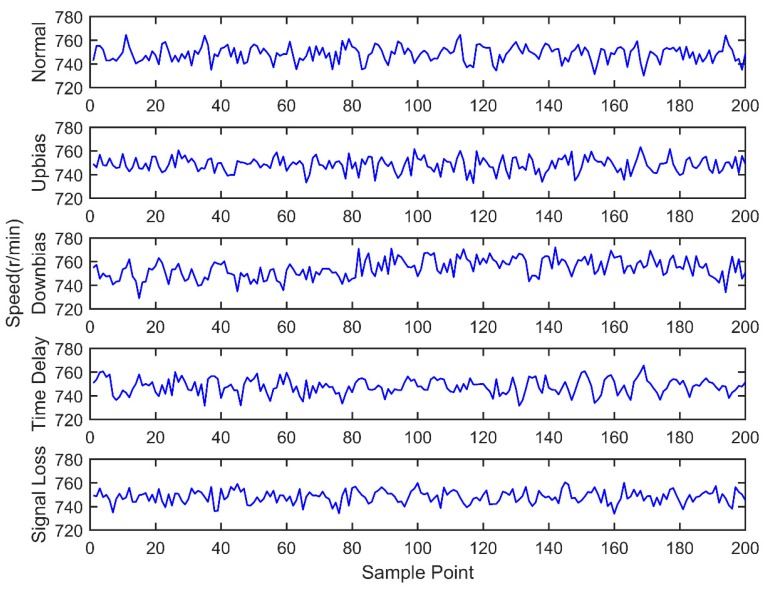
Engine speed diagram.

**Figure 12:**
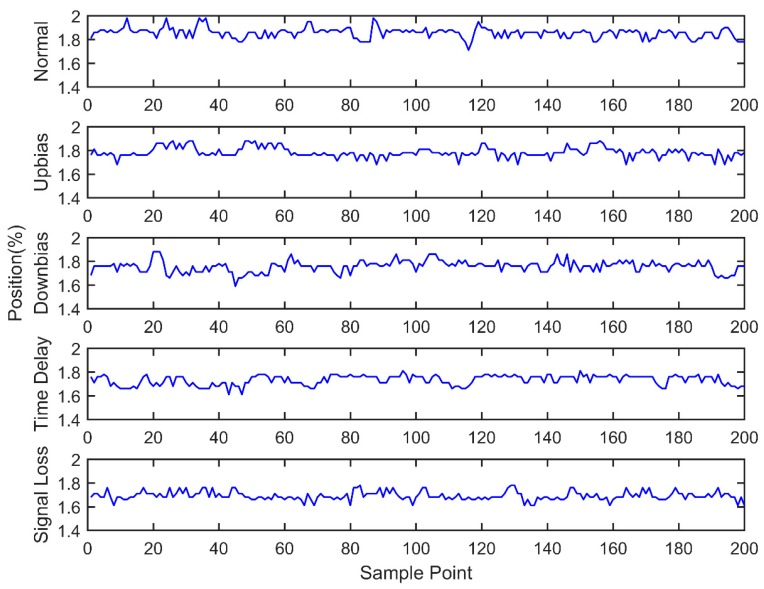
Throttle valve position diagram.

**Figure 13:**
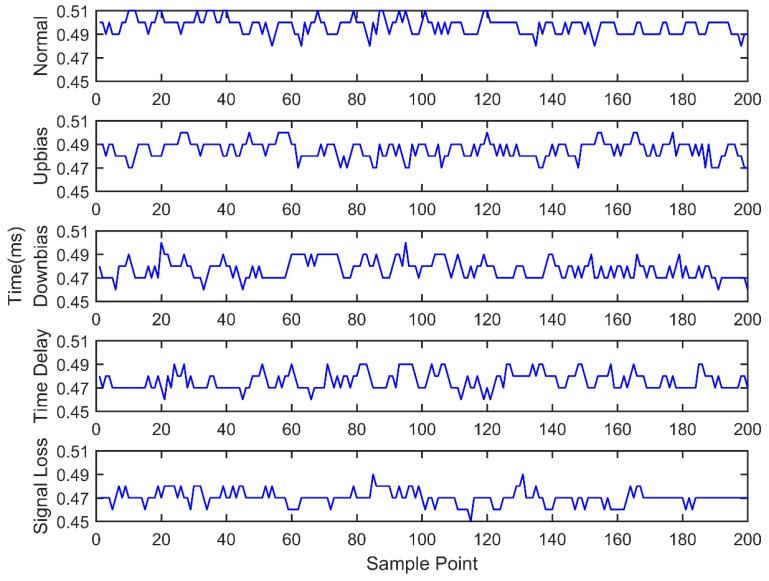
Injection time diagram.

**Figure 14:**
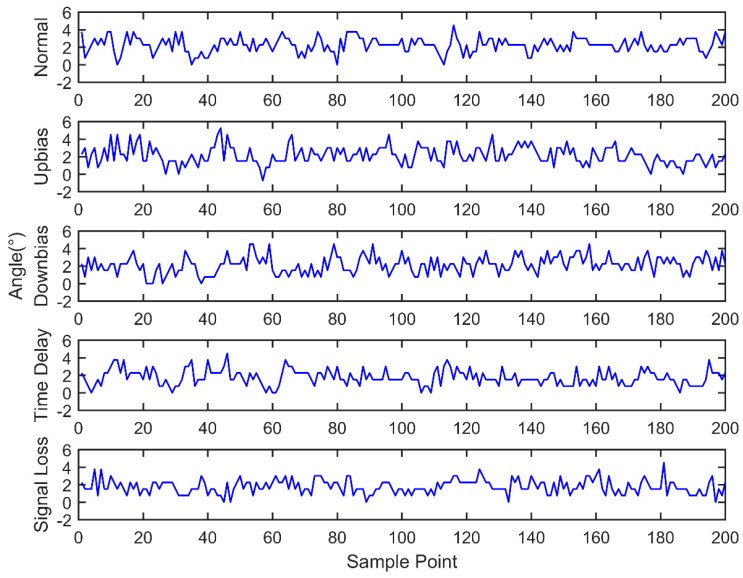
Ignition advance angle diagram.

**Figure 15:**
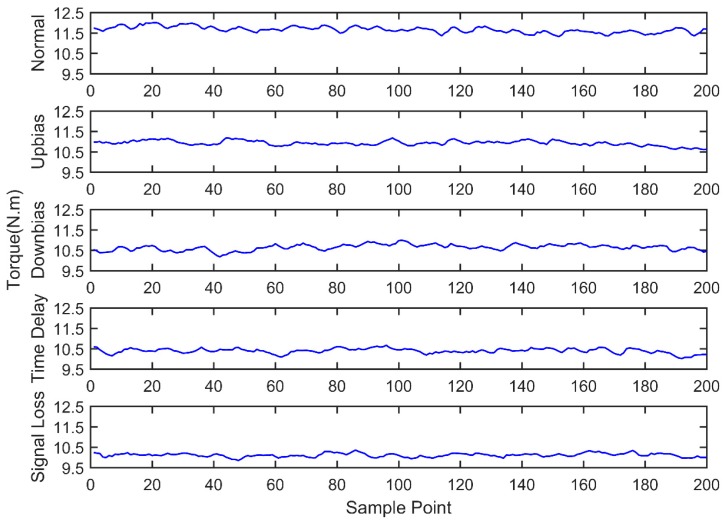
Engine indicated torque diagram.

**Figure 16:**
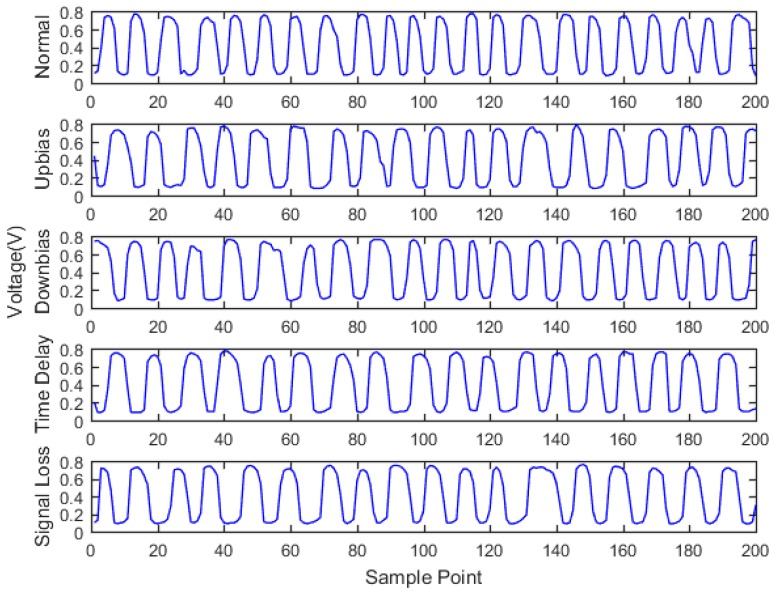
Upstream oxygen sensor voltage diagram.

At the same time, the OBD interface accesses the vehicle CAN bus, getting the indicated torque, engine speed, throttle valve position, ignition advance angle, upstream oxygen voltage, and injection time parameters with a 30 milliseconds time interval, as well as sending the acquired data to the remote monitoring platform. During the total experiment period, 200 sets of data are acquired under five fault modes, as shown in Figure 11, Figure 12, Figure 13, Figure 14, Figure 15 and Figure 16. As can be seen from the figures, it is unable to perform the fault diagnosis just according to the threshold, so it is necessary to do the fault diagnosis based on the multi information fusion.

## 5. Engine Fault Diagnosis Based on Multi Information Fusion with Experiment Data

### 5.1. Engine Fault Diagnosis on Data Fusion Layer

The neural network structure and hidden layer node numbers are determined in the previous paragraph. By comparing the fault diagnosis results of the network under different sample quantities, it shows that the higher the number of training samples, the higher the accuracy of the fault diagnosis, with the same ratio of test samples to training samples. However, more training time is needed in the test process. Table 2 is the fault diagnosis results of the BP neural network with different sample quantities. 

The accuracy calculation formula is shown as (17)

(17) $$A_{test}=N_{1}/N_{2}$$

where, $A_{test}$ is the accuracy of the diagnosis result, $N_{1}$ is the number of test samples recognized properly, and $N_{2}$ is the number of all test samples.

### 5.2. Engine Fault Diagnosis on Feature Fusion Layer

It can be found that the primary task of feature layer diagnosis is to extract feature vectors according to the established model. The experiment data of the sensor is obtained from the CAN bus with a fixed sampling frequency, which basically does not contain frequency components, so the time-domain method is suitable for its feature extraction. Feature vector parameters can be classified according to the dimension, and the dimension parameters are sensitive to the variance, while the dimensionless parameters are insensitive to the variance. These advantages and characteristics of data correlation can be combined to select seven kinds of parameters as feature vectors, including dimension parameters (mean and variance value) and dimensionless parameters (wave shape index, peak index, pulse index, margin index, and kurtosis index).

A total of 1000 data samples are acquired under five fault modes, with each mode containing 200 data samples. Then, the 200 data samples are divided into 20 parts, each part with 10 data samples. Next, the time domain feature value for each part is calculated, getting 100 sets for each fault mode in total. These data will be further randomly selected, 80 sets of each fault mode as training samples and 20 sets of each fault mode, and are sent to the SVM model to perform fault diagnosis.

**Figure 17:**
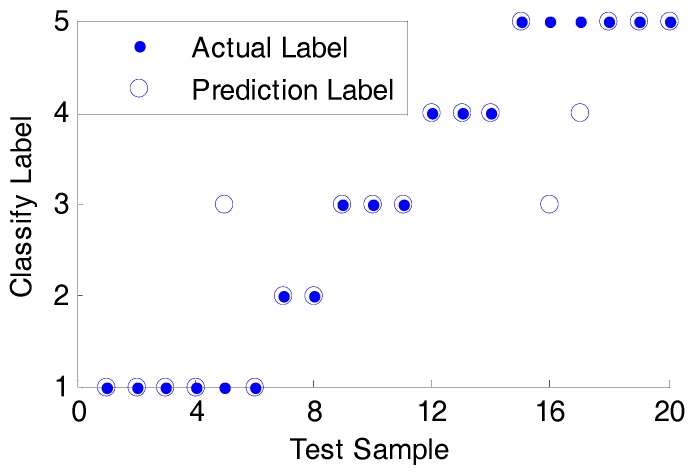
SVM diagnostic results.

Detailed classification results are shown in Figure 17. It can be found that the classification accuracy is 85% and the test validation time is 0.00907 seconds. Compared with results of the data fusion layer and feature fusion layer, there is less diagnostic time and better accuracy to some extent.

### 5.3. Engine Fault Diagnosis on Decision Fusion Layer

According to the diagnostic results of the data fusion layer and feature fusion layer, it can be found that the former has a higher accuracy, but still has the error rate and lower reliability at some extent. The latter has the contradictory situation between the forecast results and actual results after feature vectors are extracted from the acquired data. Therefore, it is necessary to use the decision layer to improve the accuracy and reliability based on the results of the data fusion layer and feature fusion layer.

#### 5.3.1. Decision Fusion Layer Based on Data Fusion Layer Diagnostic Results

In the decision fusion layer, 200 test results from the BP neural network are treated as evidence, which grouped by their true failure mode and allocated the reliability according to the D-S theory. The specific process is as follows: Firstly, the acquired data is divided into five groups according to the fault mode. Then, five copies of the results from each group are taken out as evidence and the reliability is converted. Finally, the evidence sets constituted from the diagnostic results are fused with the evidence theory. Table 3, Table 4, Table 5, Table 6 and Table 7 are typical fusion results in the five modes, where mode 1, mode 2, mode 3, mode 4, and mode 5 represent the normal signal voltage, upward bias, downward bias, time delay, and signal loss mode of the intake pressure sensor separately.

As can be seen from Table 3, Table 4, Table 5, Table 6 and Table 7, after the conversion of reliability, the evidence of each mode has a higher uncertainty, which is related to the settled reliability allocation strategy. After the decision fusion layer, the reliability of the diagnosis results is improved significantly. In mode 1, mode 2, mode 4, and mode 5, the decision layer classifies the fault modes accurately with a higher reliability. In mode 3, the diagnostic reliability is lower because the reliability of evidence 1 and evidence 3 are lower. However, after the evidence theory fusion, the reliability was improved to some extent, and misjudgment was avoided. The fusion results show that the accuracy of the data fusion layer using the BP neural network is 172/200 = 86%, and after the decision fusion layer, the diagnostic accuracy increases to 37/39 = 94.9%.

#### 5.3.2. Decision Fusion Layer Based on Feature Fusion Layer Diagnostic Results

Before the decision fusion layer, the reliability according to the vote results from the feature fusion layer are allocated. It is assumed that $M=M_{1},M_{2},\ldots ,M_{n}$ is the diagnostic classification results from *n* multiple classifiers, $C_{n}^{2}=n\times (n-1)/2$ is the sum of the output sequence classification, and the structured reliability allocation function is $m(A)=M/C_{n}^{2}=\{M_{1},M_{2},\ldots ,M_{n}\}/C_{n}^{2}$. Twenty test results from the feature fusion layer are treated as evidence and divided into five groups according to its true fault type. Table 8, Table 9, Table 10, Table 11 and Table 12 are typical fusion results for the five modes; mode1, mode 2, mode 3, mode 4, and mode 5 represent the normal signal voltage, upward bias, downward bias, time delay, and signal loss mode of the intake pressure sensor separately.

As can be seen from Table 8, Table 9, Table 10, Table 11 and Table 12, after the reliability conversion, the uncertainty reliability of the evidence is 0 in each mode, which is related to the settled reliability allocation strategy. After the decision fusion layer, the diagnostic accuracy improved significantly. In mode 1, mode 2, and mode 5, the fusion results have a higher reliability and diagnosed the fault accurately. For mode 3, the evidence quantities are small and the reliability of the fusion results is lower, however, the fault still can be diagnosed after the decision fusion. In mode 4, there are two misjudgment evidences, which cause the reliability decline, however, fault can still be diagnosed correctly. The final fusion results show that the accuracy of the feature fusion layer with SVM is 18/20 = 90%, and after the decision fusion layer, the diagnostic accuracy increases to 5/5 = 100% in the condition of the small samples.

## 6. Conclusions

Based on the analysis of the fault diagnosis methods and multi information fusion theory, this paper studies the application of the multi information fusion method to diagnose sensor faults of an engine electronic control system. The following was accomplished:

The fault diagnostic model and algorithms were studied. Based on the analysis of the characteristics of fault diagnosis and multi information fusion, a fault diagnosis model based on multi information fusion was established according to the data processing level. The model includes the data fusion layer, feature fusion layer, and decision fusion layer. The data fusion algorithm uses the BP artificial neural network, feature fusion algorithm based on the support vector machine, decision fusion algorithm based on evidence theory, and the fusion model structure, and the diagnostic process of each layer in engine fault diagnosis was settled. In the decision fusion model, based on evidence theory, the way of reliability allocation was analyzed by combining data fusion diagnosis results and feature level fusion diagnosis results as evidence.

Engine sensor fault analysis and simulator development were carried out. Based on the summary of the main simulation methods and combining the studied fault types of the electronic control system, a fault simulator was developed. The fault simulator consists of a signal switching box, AD signal acquisition module, main control unit, and DA signal output module. Under the CodeWarrior integrated development environment, fault simulator software was developed. The developed fault simulator can simulate both bias fault and drift fault of the sensor with the deal of voltage up bias, down bias, and delay.

Sensor fault diagnosis with multi information fusion was completed. Real vehicle was selected as the experimental platform. Fault diagnosis was carried out by multi information fusion with data acquired from a CAN bus. In the data layer, engine data was used to diagnosis with the BP neural network directly; in the feature layer, feature vectors were extracted from original data with the time domain method. The accuracy and training time were compared in the two fusion layers. In the decision layer, the fault diagnosis was based on evidence theory, which combines the data fusion layer and feature fusion layer results as the evidence. Diagnostic results showed that the multi information fusion method can diagnose the faults of engine electronic control systems effectively, and clearly improves the accuracy and reliability.

With the rapid development of remote diagnosis technology, car networking technology, big data technology, and promotion of T-box devices, it is easy to collect numerous process data of vehicles. However, automobile manufacturers are faced with a problem of how to use them to get what we want. For example, how to prognose the critical fault in time by analyzing the data to further ensure function safety. It is worthwhile to be researched, therefore, this will be the subject of future research.

## Figures and Tables

**Table 1:** Experiment plan.

Experiment Class	Range	Engine Condition	Sample Time
Signal Voltage Normal	N/A	Idle	120 s
Signal Voltage Upward Bias	0.36	Idle	120 s
Signal Voltage Downward Bias	0.36	Idle	120 s
Signal Voltage Time Delay	0.1 s	Idle	120 s
Signal Voltage Loss	N/A	Idle	120 s

**Table 2:** Fault diagnostic results with the BP neural network.

Sequence Number	Training Sample Numbers	Training Time(s)	Test Sample Numbers	Accuracy Rate (%)
1	800	3.7971	200	86
2	600	2.1753	150	66.7
3	400	2.3711	100	64
4	200	1.2884	50	52

**Table 3:** Decision fusion layer results in mode 1 based on the data layer.

Evidence	Mode 1 Reliability	Mode 2 Reliability	Mode 3 Reliability	Mode 4 Reliability	Mode 5 Reliability	Uncertainty Reliability
1	0.4689	0.0262	0.0672	0.0014	0.0057	0.4305
2	0.4687	0.0250	0.0697	0.0015	0.0042	0.4310
3	0.4689	0.0254	0.0693	0.0014	0.0042	0.4309
4	0.4689	0.0254	0.0692	0.0014	0.0042	0.4308
5	0.4690	0.0155	0.0790	0.0021	0.0021	0.4323
Fusion Result	0.9390	0.0074	0.0277	0.0004	0.0012	0.0243
Expected Result	1	0	0	0	0	0

**Table 4:** Decision fusion layer results in mode 2 based on the data layer.

Evidence	Mode 1 Reliability	Mode 2 Reliability	Mode 3 Reliability	Mode 4 Reliability	Mode 5 Reliability	Uncertainty Reliability
1	0.0014	0.4501	0.0826	0.0041	0.0032	0.4587
2	0.0014	0.4546	0.0764	0.0041	0.0064	0.4570
3	0.0012	0.4356	0.0939	0.0039	0.0034	0.4621
4	0.0012	0.4329	0.0956	0.0039	0.0037	0.4627
5	0.0017	0.4649	0.0715	0.0042	0.0029	0.4548
Fusion Result	0.0005	0.9232	0.0418	0.0014	0.0014	0.0318
Expected Result	0	1	0	0	0	0

**Table 5:** Decision fusion layer results in mode 3 based on the data layer.

Evidence	Mode 1 Reliability	Mode 2 Reliability	Mode 3 Reliability	Mode 4 Reliability	Mode 5 Reliability	Uncertainty Reliability
1	0.0007	0.2790	0.2277	0.0064	0.0079	0.4784
2	0.0006	0.2217	0.2828	0.0084	0.0072	0.4793
3	0.0009	0.2726	0.2408	0.0028	0.0027	0.4801
4	0.0006	0.1929	0.3213	0.0014	0.0052	0.4786
5	0.0002	0.0506	0.5228	0.0104	0.0081	0.4079
Fusion Result	0.0003	0.2581	0.6794	0.0037	0.0038	0.0546
Expected Result	0	0	1	0	0	0

**Table 6:** Decision fusion layer results in mode 4 based on the data layer.

Evidence	Mode 1 Reliability	Mode 2 Reliability	Mode 3 Reliability	Mode 4 Reliability	Mode 5 Reliability	Uncertainty Reliability
1	0.0002	0.0690	0.0754	0.4404	0.0172	0.3978
2	0.0002	0.0224	0.1450	0.4113	0.0058	0.4153
3	0.0002	0.0200	0.1096	0.3116	0.1323	0.4263
4	0.0002	0.0206	0.1286	0.4326	0.0109	0.4070
5	0.0000	0.0316	0.1060	0.4387	0.0217	0.4019
Fusion Result	0.0001	0.0135	0.0680	0.8752	0.0144	0.0289
Expected Result	0	0	0	1	0	0

**Table 7:** Decision fusion layer results in mode 5 based on the data layer.

Evidence	Mode 1 Reliability	Mode 2 Reliability	Mode 3 Reliability	Mode 4 Reliability	Mode 5 Reliability	Uncertainty Reliability
1	0.0002	0.0229	0.0696	0.0366	0.4510	0.4197
2	0.0002	0.0225	0.0706	0.0375	0.4490	0.4202
3	0.0002	0.0234	0.0682	0.0328	0.4569	0.4185
4	0.0002	0.0226	0.0703	0.0359	0.4512	0.4198
5	0.0002	0.0221	0.0729	0.0567	0.4240	0.4240
Fusion Result	0.0001	0.0076	0.0297	0.0145	0.9226	0.0255
Expected Result	0	0	0	0	1	0

**Table 8:** Decision fusion layer results in mode 1 based on the feature layer.

Evidence	Mode 1 Reliability	Mode 2 Reliability	Mode 3 Reliability	Mode 4 Reliability	Mode 5 Reliability	Uncertainty Reliability
1	0.4	0.2	0.3	0	0.1	0
2	0.4	0.2	0.3	0	0.1	0
3	0.4	0.2	0.3	0	0.1	0
4	0.4	0.1	0.3	0	0.2	0
5	0.4	0.1	0.3	0	0.2	0
Fusion Result	0.800625	0.006255	0.189992	0	0.003127	0
Expected Result	1	0	0	0	0	0

**Table 9:** Decision fusion layer results in mode 2 based on the feature layer.

Evidence	Mode 1 Reliability	Mode 2 Reliability	Mode 3 Reliability	Mode 4 Reliability	Mode 5 Reliability	Uncertainty Reliability
1	0.1	0.4	0.2	0.1	0.2	0
2	0	0.4	0.3	0.2	0.1	0
3	0.1	0.4	0.3	0	0.2	0
4	0.1	0.4	0	0.2	0.3	0
Fusion Result	0	0.955224	0	0	0.044776	0
Expected Result	0	1	0	0	0	0

**Table 10:** Decision fusion layer results in mode 3 based on the feature layer.

Evidence	Mode 1 Reliability	Mode 2 Reliability	Mode 3 Reliability	Mode 4 Reliability	Mode 5 Reliability	Uncertainty Reliability
1	0.1	0	0.4	0.2	0.3	0
2	0.3	0	0.4	0.1	0.2	0
Fusion Result	0.111111	0	0.592593	0.074074	0.222222	0
Expected Result	0	0	1	0	0	0

**Table 11:** Decision fusion layer results in mode 4 based on the feature layer.

Evidence	Mode 1 Reliability	Mode 2 Reliability	Mode 3 Reliability	Mode 4 Reliability	Mode 5 Reliability	Uncertainty Reliability
1	0.3	0.1	0	0.2	0.4	0
2	0.2	0.3	0	0.4	0.1	0
3	0.1	0.4	0	0.2	0.3	0
4	0.1	0.2	0	0.4	0.3	0
5	0.1	0.3	0	0.4	0.2	0
Fusion Result	0.014778	0.17734	0	0.630542	0.17734	0
Expected Result	0	0	0	1	0	0

**Table 12:** Decision fusion layer results in mode 5 based on the feature layer.

Evidence	Mode 1 Reliability	Mode 2 Reliability	Mode 3 Reliability	Mode 4 Reliability	Mode 5 Reliability	Uncertainty Reliability
1	0.2	0.1	0	0.3	0.4	0
2	0.2	0.1	0	0.3	0.4	0
3	0.1	0.2	0	0.3	0.4	0
4	0.3	0.1	0	0.2	0.4	0
Fusion Result	0.037037	0.006173	0	0.166667	0.790123	0
Expected Result	0	0	0	0	1	0

## References

[1] Zhang J., Yao H., Rizzoni G. (2017). Fault diagnosis for electric drive systems of electrified vehicles based on structural analysis. IEEE Trans. Veh. Technol..

[2] Zhang J. (2016). Model-Based Fault Diagnosis For Automotive Functional Safety. Doctor’s Thesis.

[3] Rizzoni G., Azzoni P.M., Minelli G. On-board diagnosis of emission control system malfunctions in electronically controlled spark ignition engines. Proceedings of the American Control Conference IEEE.

[4] Blanke M. (2006). Diagnosis and Fault-Tolerant Control.

[5] Simani S., Fantuzzi C., Patton R.J. (2013). Model-Based Fault Diagnosis Techniques.

[6] Yin S. (2014). Data-Driven Design of Fault Diagnosis Systems.

[7] Yin S., Wang G., Karimi H.R. (2014). Data-driven design of robust fault detection system for wind turbines. Mechatronics.

[8] Jia F. (2018). Deep normalized convolutional neural network for imbalanced fault classification of machinery and its understanding via visualization. Mech. Syst. Signal Process..

[9] Carbot-Rojas D.A., Escobar-Jiménez R.F., Gómez-Aguilar J.F. (2017). A survey on modeling, biofuels, control and supervision systems applied in internal combustion engines. Renew. Sustain. Energy Rev..

[10] Zhou J., Xu L. The fault diagnosis of marine engine cooling system based on artificial neural network (ANN). Proceedings of the International Conference on Computer and Automation Engineering 2010.

[11] Guo D., Liu H., Li R., Zhang Y. Application of BP network on fault diagnosis of a truck engine. Proceedings of the 2016 IEEE Information Technology, Networking, Electronic and Automation Control Conference.

[12] Glowacz A. (2018). Acoustic based fault diagnosis of three-phase induction motor. Appl. Acoust..

[13] Tadeusiewicz R. (2015). Neural networks in mining sciences-general overview and some representative examples. Arch. Min. Sci..

[14] Ganovska B. (2016). Design of the model for the on-line control of the AWJ technology based on neural networks. Indian J. Eng. Mater. Sci..

[15] Pan R., Lin X. The application of support vector machine on fault diagnosis of the diesel engine exhaust gas turbocharger. Proceedings of the International Conference on Computer Distributed Control and Intelligent Environmental Monitoring.

[16] Li Y., Yang Y., Li G., Xu M., Huang W. (2017). A fault diagnosis scheme for planetary gearboxes using modified multi-scale symbolic dynamic entropy and mRMR feature selection. Mech. Syst. Signal Process..

[17] Yao Z., Pan H. Engine fault diagnosis based on the weighted DS evidence theory. Proceedings of the 2014 IEEE International Workshop on Computational Intelligence and Applications.

[18] Zhang Y., Zhang C., Sun J., Guo J. (2018). Improved wind speed prediction using empirical mode decomposition. Adv. Electr. Comput. Eng..

[19] Agaram V. (2014). Reliability of multi-sensor fusion for next generation cars and trucks. SAE Tech. Pap..

[20] Moosavian A., Khazaee M., Najafi G. (2015). Spark plug fault recognition based on sensor fusion and classifier combination using dempster–shafer evidence theory. Appl. Acoust..

[21] Basir O., Yuan X. (2007). Engine fault diagnosis based on multi-sensor information fusion using dempster–shafer evidence theory. Inf. Fusion.

[22] Gao X. (2008). Research on Fault Diagnosis of Diesel Engine Based on Multi Sensor Information Fusion. Master’s Thesis.

[23] Wu W. (2011). Fault Diagnosis Method of Aero Engine Based on Information Fusion. Master’s Thesis.

[24] Zhang L. (2009). Research on Fault Diagnosis Method of Gasoline Engine Electronic Control System Based on Information Fusion. Master’s Thesis.

[25] Kai C. (2013). Research on Light-Duty Gasoline Vehicle Remote Monitoring and Fault Diagnosis Technology. Master’s Thesis.

[26] Wen D., Wu X.H., Ling D., Chen Z.Z., Wang H. Application of dempster-shafer evidence theory in fault diagnosis of aero-engine gas path. Proceedings of the International Conference on Quality, Reliability, Risk, Maintenance, and Safety Engineering.

[27] Wang Z. (2001). Research and Application of Fault Diagnosis Method Based on Information Fusion Technology. Master’s Thesis.

[28] Zhang L., Tao J. (2018). Research on degeneration model of neural network for deep groove ball bearing based on feature fusion. Algorithms.

